# TCGA Pan-Cancer Genomic Analysis of Alternative Lengthening of Telomeres (ALT) Related Genes

**DOI:** 10.3390/genes11070834

**Published:** 2020-07-21

**Authors:** Isaac Armendáriz-Castillo, Andrés López-Cortés, Jennyfer García-Cárdenas, Patricia Guevara-Ramírez, Paola E. Leone, Andy Pérez-Villa, Verónica Yumiceba, Ana K. Zambrano, Santiago Guerrero, César Paz-y-Miño

**Affiliations:** 1 Centro de Investigación Genética y Genómica, Facultad de Ciencias de la Salud Eugenio Espejo, Universidad UTE, 170129 Quito, Ecuador; fabian.armendariz@ute.edu.ec (I.A.-C.); aalc84@gmail.com (A.L.-C.); jennyfer.garcia@ute.edu.ec (J.G.-C.); alexandra.guevara@ute.edu.ec (P.G.-R.); paola.leone@ute.edu.ec (P.E.L.); andy.perez@ute.edu.ec (A.P.-V.); veronica.yumiceba@ute.edu.ec (V.Y.); anak.zambrano@ute.edu.ec (A.K.Z.); 2 Latin American Network for Implementation and Validation of Clinical Pharmacogenomics Guidelines (RELIVAF-CYTED), Madrid, Spain

**Keywords:** telomeres, cancer, ALT, in silico

## Abstract

Telomere maintenance mechanisms (TMM) are used by cancer cells to avoid apoptosis, 85–90% reactivate telomerase, while 10–15% use the alternative lengthening of telomeres (ALT). Due to anti-telomerase-based treatments, some tumors switch from a telomerase-dependent mechanism to ALT; in fact, the co-existence between both mechanisms has been observed in some cancers. Although different elements in the ALT pathway are uncovered, some molecular mechanisms are still poorly understood. Therefore, with the aim to identify potential molecular markers for the study of ALT, we combined in silico approaches in a 411 telomere maintenance gene set. As a consequence, we conducted a genomic analysis of these genes in 31 Pan-Cancer Atlas studies from The Cancer Genome Atlas and found 325,936 genomic alterations; from which, we identified 20 genes highly mutated in the cancer studies. Finally, we made a protein-protein interaction network and enrichment analysis to observe the main pathways of these genes and discuss their role in ALT-related processes, like homologous recombination and homology directed repair. Overall, due to the lack of understanding of the molecular mechanisms of ALT cancers, we proposed a group of genes, which after ex vivo validations, could represent new potential therapeutic markers in the study of ALT.

## 1. Introduction

Telomeres are nucleoprotein complexes that consist of a tandem 5’-TTAGGG-3’ sequence and protect the ends of eukaryotic chromosomes, preventing DNA damage response (DDR), end-to-end fusions and genomic instability [1]. Telomeric DNA ranges from 3 to 15 Kb in humans, leaving a G-rich 3’-single-strand extended beyond the complementary chain, usually called the G-overhang [1,2]. To avoid the end-protection problem due to the overhang chain, the G-tail folds into itself, invading the double-stranded telomeric DNA, forming a lasso-like structure called the t-loop [3], which is protected by a six protein complex called shelterin [4], essential for telomere replication and regulation [2,3]. The shelterin complex is composed of the telomeric repeat binding factors 1 and 2 (TRF1 and TRF2), which interact with the telomeric double-stranded DNA and the protection of telomeres 1 (POT1) that bind to the single-stranded DNA [5].

TRF1 and TRF2 associate directly with the TRF1 interacting nuclear factor 2 (TINF2 or TIN2), which stabilizes their bond with the telomere; the shelterin complex subunit, the telomerase recruiting factor (ACD or TPP1) and the TRF2 interacting protein (TRF2IP or RAP1) complete the complex that protects and regulates telomere structure, DNA damage response and lengthening [5]. Nonetheless, with each round of the cell cycle, telomeres lose about 200 nucleotides; and eventually, this shortening leads to senescence or apoptosis [6].

To avoid senescence or apoptosis caused by telomere shortening, cancer cells take advantage of a set of mechanisms known as Telomere Maintenance Mechanisms (TMM), which include: telomerase reactivation and the alternative lengthening of telomeres (ALT) [1,6]. A high proportion of tumors reactivate the expression of telomerase to maintain its chromosomal ends, while 10–15% of human cancers use the ALT pathway [7]. ALT is frequently detected in tumors of mesenchymal or neuroephitelial origin [8], but there have been reports of the ALT mechanism detected in a fraction of epithelial tumors like: breast, skin, ovarian, uterus and gastric, among others [3,9].

ALT-positive (ALT+) cells display a characteristic phenotype. Telomeric DNA in ALT+ cells is constantly elongating, commonly depending on break-induced replication (BIR) processes, which are part of the homologous recombination (HR) pathway or homology-directed repair (HDR) mechanism [10,11]. ALT+ cells also show heterogenous telomere length, abundant extrachromosomal repeats (ECTRs), telomere sister chromatid exchange (T-SCE) [12] and high levels of extrachromosomal single-stranded telomeric DNA known as c-circles, which are molecular markers for ALT+ cells detection [13].

The main characteristic of ALT+ telomeres is the interaction with promyelocytic leukemia (PML) proteins, which altogether are believed to function as platforms for telomere recombination and are called ALT-associated PML bodies (APBs) [12,14]. In fact, there is evidence that the disruption of APBs blocks the ALT mechanism [12].

Although, many ALT-related genomic alterations have been resolved, such as the loss of function of *ATRX, DAXX, H3F3A*, or the BIR-related pathways dependent or independent of *RAD51* and *RAD52* [1,7,10,15], the molecular basis through which ALT occurs remains elusive and poorly understood [7]

Many anti-telomerase-based cancer therapies are believed to cause the switching in some tumors from telomerase to ALT [3,16]. Indeed, the co-existence of both telomerase-dependent and ALT mechanisms have been reported in different cancer types [3,17]. This evidence sheds light on ALT as a potential target for therapy, because of the poor prognosis associated with these cancer types [1,7,18]. Different drugs have been tested in the last years to target ALT+ cells, however, most of them have been unsuccessful and still are in phases 1/2 of clinical trials [1,12].

Even though several ALT-associated factors have been uncovered, the pathway activation, regulation and functioning require further investigation [12]. Therefore, the identification of new potential molecular markers would be of enormous importance in the future design of new strategies for the detection and treatment of ALT+ cancers.

To fulfill this need, we evaluated the genomic alterations of 411 telomere maintenance (TM) genes in 9282 samples from 31 Pan-Cancer Atlas (PCA) studies and applied different in silico approaches with the aim to identify and propose a set of genes which after ex-vivo validations could be used as potential molecular markers to improve the study of the ALT pathway.

## 2. Materials and Methods

### 2.1. Gene Set

TelNet (http://www.cancertelsys.org/telnet/) is a database that groups more than 2000 human TM genes. All genes are annotated according to their classification of TMM, TM function and a significance score given by the evidence of gene function in telomeres [19]. The database shows the role of each gene in ALT and telomerase-mediated mechanisms and classifies them as enhancers, repressors or ambiguous (when their role in the ALT mechanism is defined but their activity is not yet clear). Therefore, the TelNet database was downloaded and manually filtered in order to select a set of genes that were associated with ALT activities as: enhancers, repressors and ambiguous. Consequently, a total of 411 TM genes were selected for the study.

### 2.2. Genomic Alterations

Genomic alterations (CNV amplification, CNV deep deletion, in-frame mutation, truncating mutation, missense mutation, fusions, mRNA up and down regulation) were analyzed in the cBio Portal (http://www.cbioportal.org/) [20,21]. A total of 9282 samples were selected from 31 Pan-Cancer Atlas (PCA) studies from The Cancer Genome Atlas (TCGA) (LAML, ACC, BLCA, LGG, BRCA, CESC, CHOL, COAD, DLBC, ESCA, GBM, HNSC, KIRC, KIRP, LIHC, LUAD, LUSC, MESO, OV, PAAD, PCPG, PRAD, SARC, SKCM, STAD, TGCT, THYM, THCA, UCS, UCEC and UVM) [22,23,24,25,26,27,28,29,30,31]. The frequency means of genomic alterations were compared with a Bonferroni correction test (*p*< 0.05) by using SPSS Statistics Software (IBM Corp., Armonk, NY, USA).

### 2.3. Prevalence of the ALT Mechanism in Different Cancer Types

The ALT mechanism is frequent in tumors of mesenchymal origin; however, ALT positive cells have been observed in epithelial tumors. Therefore, the prevalence of the ALT mechanism in the different TCGA cancer studies was reported as part of this study and the tumors were classified in three different categories: frequent ALT tumors, rare ALT tumors and not reported (for tumors without evidence of ALT+ cells). Additionally, the first quartile of the most altered genes from the PCA studies was classified in each of the categories described earlier.

### 2.4. Protein-Protein Interaction Network

In order to predict the interactions among the ALT-related proteins, the STRING database (https://string-db.org/) was used. An interaction score of 0.9 (highest confidence) was set in the system configuration [32,33] and the most significant signaling pathways (*p*< 0.001) related to ALT were selected and differentiated by colors in the network.

Break-induced replication (BIR) is a main process used by ALT+ cells to extend their telomeres [34], therefore, the gene set of the homologous recombination pathway was downloaded from the Kyoto Encyclopedia of Genes and Genomes (KEGG) [35] and an interaction network with the ALT-related genes selected in this study was constructed with the aid of the STRING database; an interaction score of 0.9 (highest confidence) was set in the system configuration. Proteins interactions were determined according to database curation, experimental determination and co-expression [36].

### 2.5. Gene Set Enrichment Analysis

An enrichment analysis was made using the g:Profiler tool (https://biit.cs.ut.ee/gprofiler/gost) [37]. The most significant pathways were selected after Benjamini–Hochberg and False Discovery Rate (FDR) corrections (*p*< 0.001), based on Gene Ontology (GO), Molecular Function (MF), Biological Process (BP), KEGG, REACTOME database and Human Phenotype (HP).

## 3. Results

### 3.1. Role of the TM Gene Set in the ALT Mechanism

**Figure 1:**
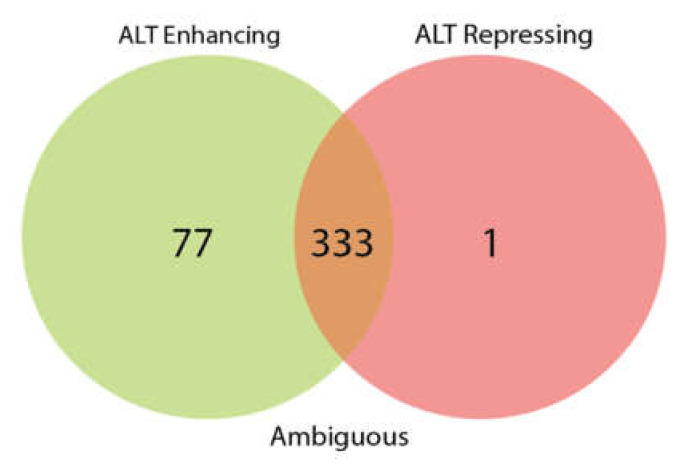
Role of the gene set in the ALT mechanism. The 411 gene set selected for this study was filtered according of their alternative lengthening of telomeres (ALT) activity in the TelNet database and classified as enhancers, repressors or ambiguous.

The 411 genes from the TelNet database [19] (Table S1) were filtered according to their activity in ALT. Figure 1 shows a Venn diagram with the reported TMM activity of the gene set; 77 genes were classified as enhancers of ALT, 1 as repressor and 333 had ambiguous activity.

### 3.2. Genomic Alterations

To address the genomic alterations of ALT-related genes, these were analyzed in the cBioPortal [20,21] by selecting 9282 samples from 31 PCA studies from TCGA [22,23,24,25,26,27,28,29,30,31] (Table 1).

**Figure 2:**
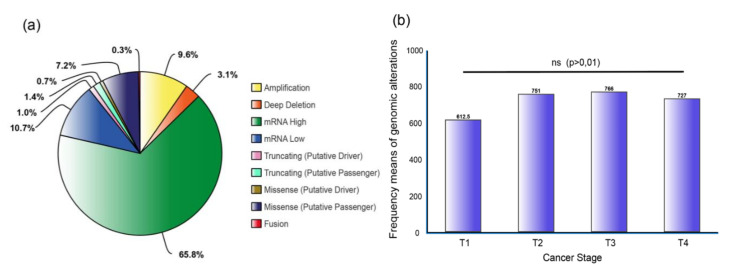
Genomic alterations. (**a**) Percentage of genomic alterations of 411 ALT-related genes distributed in the 31 PCA studies. (**b**) Frequency means of genomic alterations of the 411 genes in different cancer stages; no significant difference was observed after Bonferroni correction test (*p* > 0.01). All values were normalized by the number of samples in each stage.

A total of 325,936 genomic alterations were identified (Table S2) and a pie chart with the most frequent alterations was constructed after all values were normalized by the number of samples. Figure 2a shows mRNA upregulation at the top with 65.8%, followed by mRNA downregulation (10.7%), copy number alterations (CNA): amplifications (9.6%), missense mutation (putative passenger) with 7.2%, deep deletion (3.1%) and truncating mutations and fusion genes with less than 2%.

Finally, to better understand the implication of ALT-related genes in cancer progression, from primary tumors to metastasis, we analyzed the genomic alterations in each cancer stage (Figure 2b) (Table S3). However, no significant difference was observed after a Bonferroni correction test (*p* > 0.01). As a result, it can be inferred that TM genes alterations and the ALT pathway across different types of tumors are not dependent on cancer staging.

### 3.3. TM Genes Validation and TCGA Pan-Cancer Studies Frequencies

**Figure 3:**
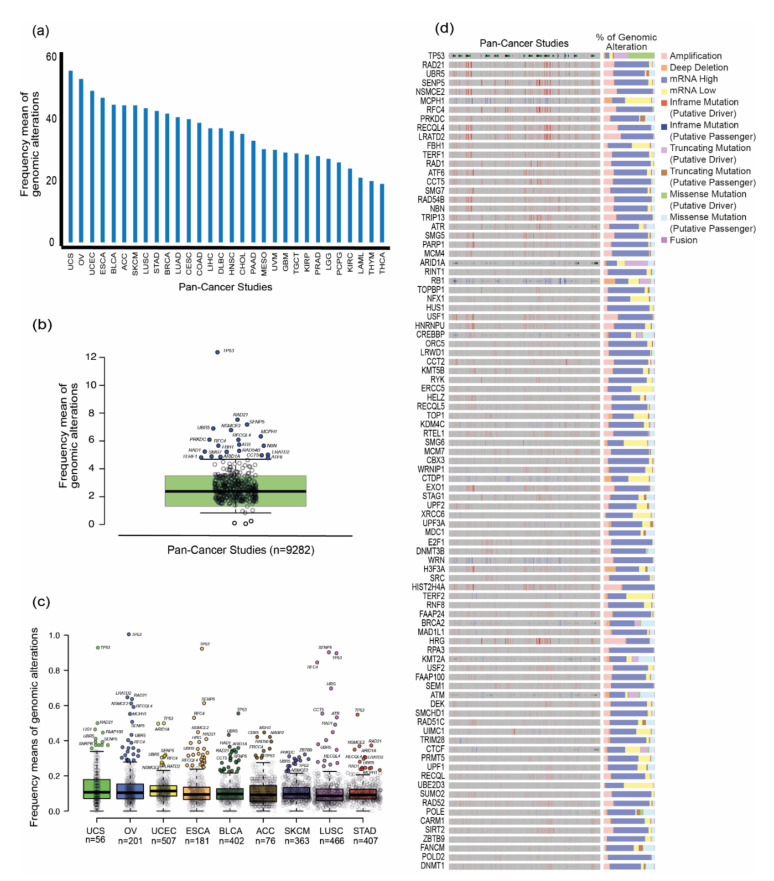
Top altered cancers and oncoprint. (**a**) Shows the most altered cancers according to the frequency means of genomic alterations of the 411 genes previously normalized by the number of samples in each PCA study. (**b**) Shows a boxplot with frequency means of genomic alterations of the 411 genes in all the 31 PCA studies; 20 genes were identified as highly altered and are represented in the blue dots. (**c**) Shows boxplots of the 411 genes with each of the first quartiles of the most altered PCA studies. The unusual altered genes are represented in color dots per each cancer study. (**d**) Shows the oncoprint of the most altered genes across the PCA studies, with the individual genomic alteration’s profile of each gene marked in different colors.

The gene set and each TCGA study were ordered by the highest frequency mean of genomic alterations to the lowest (Table S4). To narrow down our analysis, the first-quartile of genes (*n* = 103) was selected for further analysis and the highest frequencies of alterations for the 31 PCA studies were: UCS (56.054), OV (53.194), UCEC (49.387), ESCA (47.088), BLCA (44.883), ACC (44.658), SKCM (44.620), LUSC (43.822), STAD (43.005), BRCA (42.068), LUAD (40.942), CESC (40.116), COAD (39.038), SARC (37.418), LIHC (37.353), DLBC (37.256), HNSC (36.381), CHOL (35.444), PAAD (33.256), MESO (30.634), UVM (30.225), GBM (29.366), TGCT (29.132), KIRP (28.708), PRAD (28.258), LGG (27.387), PCPG (26.292), KIRC (24.168), LAML (21.255), THYM (20.185), and THCA (19.319) (Figure 3a).

To further identify which genes displayed abnormal values of genomic alterations in the 31 PCA studies, a boxplot with these frequencies was constructed for the 411 ALT-related genes (Figure 2b). Hence, after boxplot analysis, 20 genes showed an unusual value of genomic alterations; these were selected and proposed as candidate genes to be studied in the ALT pathway: *TP53* (12.370)*, RAD21* (7.529)*, SENP5* (7.179)*, UBR5* (6.897)*, NSMCE2* (6.779)*, MCPH1* (6.332)*, PRKDC* (6.091)*, RFC4* (5.662)*, RECQL4* (5.656)*, NBN* (5.587)*, FBH1* (5.239)*, RAD1* (5.221)*, CCT5* (5.126)*, LRATD2* (5.013)*, RAD54B* (4.969)*, ATR* (4.940)*, SMG7* (4.888)*, TERF1* (4.817)*, ATF6* (4.778), and *ARID1A* (4.357).

Then, to obtain insights of the most altered genes in the PCA studies, additional boxplots were constructed (Figure 3c), along with the alteration frequencies of the 411 genes and the first quartile of the PCA studies with the highest means of genomic alterations (UCS, OV, UCEC, ESCA, BLCA, ACC, SKCM, LUSC and STAD). On this basis, the following genes were identified to be highly altered in more than one of the nine types of tumors: *TP53* (9)*, UBR5* (8)*, RAD21* (6)*, SENP5* (6)*, NSMCE2* (5)*, RFC4* (5)*, MCPH1* (4)*, RECQL4* (4)*, ARID1A* (3)*, CCT5* (3)*, LRATD2* (3), and *RAD1* (3).

Finally, to elucidate which genomic alteration is most frequently observed across the 31 PCA studies, an oncoprint with the percentage of each alteration is shown for the first-quartile of genes (Figure 3d). As expected, mRNA upregulation is the most common alteration in the gene set, with certain exceptions where amplifications, mRNA downregulation and truncating mutations, appear most frequently.

### 3.4. Prevalence of the ALT Mechanism in the TCGA PanCancer tumors

**Figure 4:**
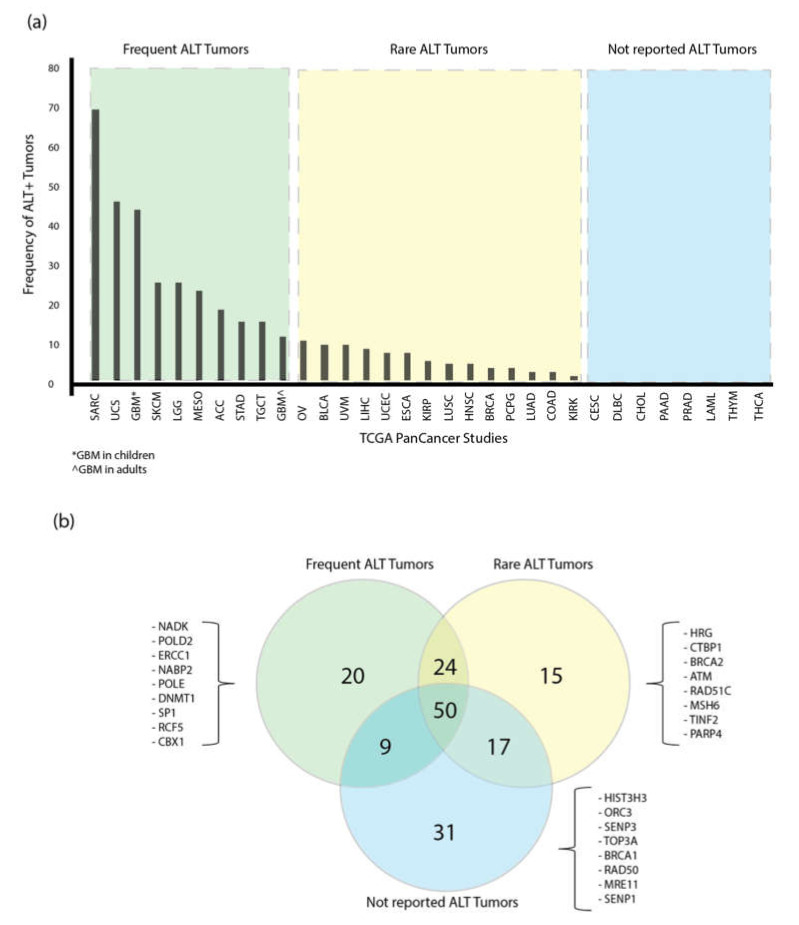
ALT mechanism prevalence in the PCA studies and its relationship with TM altered genes. (**a**) Shows the prevalence of the ALT mechanism in the 31 PCA studies; tumors were classified in three groups depending on the values above the mean as: Frequent ALT tumors, rare ALT tumors and not reported. (**b**) A Venn diagram showing the distribution of the most altered ALT-related genes in each ALT tumor group.

The presence of ALT in tumors across the 31 PCA studies have been analyzed and classified in three different categories: ALT frequent tumors, for cancers with ALT prevalence above the mean, ALT rare tumors, for cancers with prevalence below the mean, and tumors with no reports of the presence of ALT (Figure 4).

The 20 most altered genes across the 31 PCA studies are shown in Figure 3b. Furthermore, with the aim to classify the first-quartile of the highest mutated ALT-related genes (*n* = 103) in the PCA studies in each ALT tumor group, a Venn diagram was constructed (Figure 4b). As a result, a total of 20 genes was reported to be altered exclusively in the group of frequent ALT tumors, 15 genes in the rare ALT tumors group and 31 in the tumors with any reports of ALT. The overlapped areas in the Venn diagram show the number of altered genes shared among the groups.

As shown in Figure 4b, there are 24 genes shared between frequent and rare ALT tumors, and 50 genes are shared among the three groups; all 20 ALT-related genes (100%) proposed in this study are highly mutated in the PCA studies (Figure 3b) and included in the 50 genes list; according to these results, the frequency of alterations of ALT-related genes is distributed among all cancer types. In contrast, any of the 20 ALT-related genes proposed are included in the 31 genes of the not-reported ALT tumors group. This result matches with our previous analysis from the boxplots and supports our selection of the 20 genes as potential markers for the study of ALT+ tumors. This analysis will allow to focus in a particular set of genes in future ex vivo validations in a selected group of tumors. A random representation of the most altered ALT-related genes for each group is shown in Figure 4b, and the complete list of the genes included in each ALT tumor type is shown in Table S6.

### 3.5. Protein-protein interaction (PPi) Network and Enrichment Analysis

**Figure 5:**
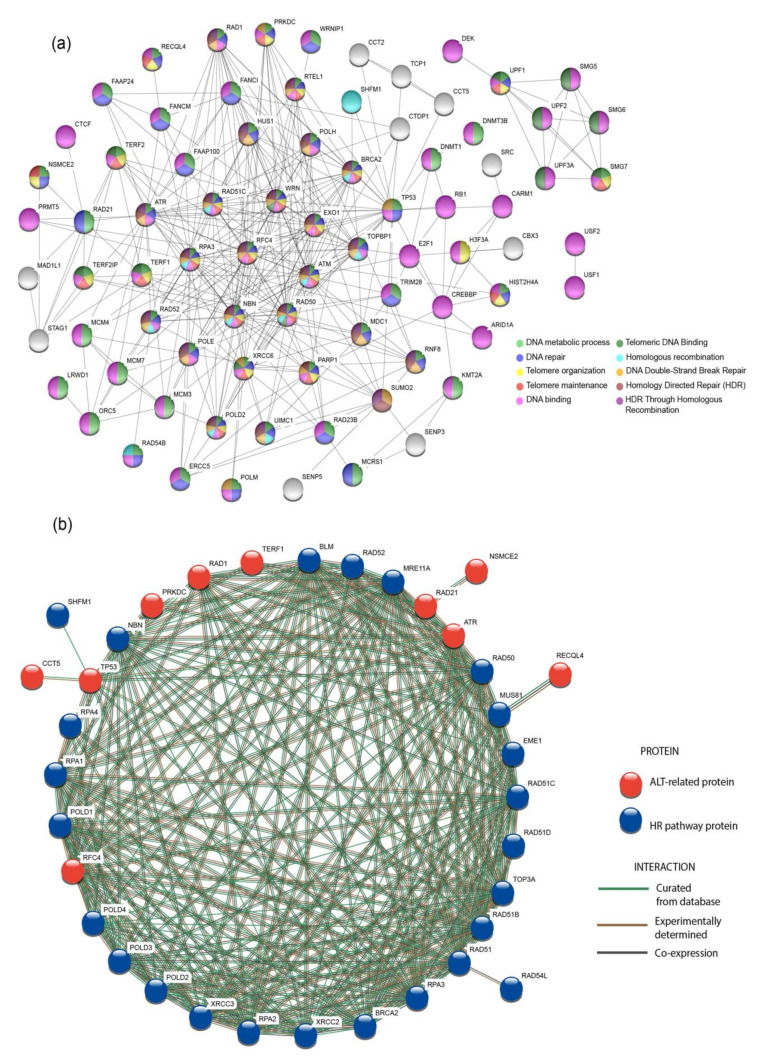
Protein-protein interaction (PPi) network and BIR-related pathway. (**a**) Shows the PPi network constructed in the STRING database; the most significant pathways are marked with different colors and represented in the nodes. (**b**) Shows the interaction network of the BIR-related pathway with the potential ALT targets selected in this study; protein interactions are classified according to database curation, experimental determination and co-expression. HR: Homologous Recombination.

PPi networks are useful resources to understand how proteins interact between them in the cell [38], hence, the STRING database [32] was used to observe the interactions of 103 proteins. By using an interaction score of highest confidence (0.9) [33], a network was constructed; additionally, the most significant pathways (*p*< 0.001) were selected and marked with different colors in the nodes (Figure 5a). As a result, 68% of the proteins in the network are essential for DNA binding; 59% for DNA metabolic process; 47% for DNA repair; 23% for telomere organization and maintenance; 12% for telomeric DNA binding; and 22% are involved in DNA Double-Strand Break Repair, HDR and HDR through Homologous Recombination (HRR).

ALT+ tumors are known to use a BIR-related process for telomere extension. In consequence, the proteins from the HR pathway were analyzed with the aid of a PPi network with the 20 proteins selected as potential ALT targets in this study by following the same criteria used for constructing the network in Figure 5a. These interactions are shown in Figure 5b; as a consequence, 10 out of 20 proteins (RAD1, TERF1, RAD21, ATR, RFC4, TP53, PRKDC, CCT5, NSMCE2, and RECQL4) showed interactions with the BIR pathway; it is evident how the ALT-related proteins are co-expressing with the proteins from the BIR pathway; the relevance of the results will be discussed later.

**Figure 6:**
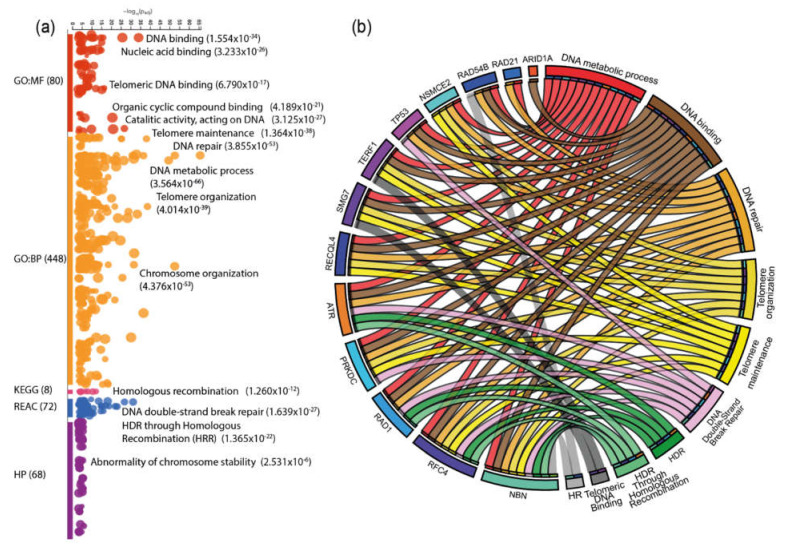
Enrichment analysis and protein-pathway correlation. (**a**) Shows the enrichment profile of 103 proteins; the most significant pathways for MF, BP, KEGG, Reactome, and HP are also shown next to the dots with its *p*-values. (**b**) Shows a CIRCOS plot corelating the genes with the highest means of alterations with its most significant pathways from the protein enrichment analysis. KEGG: Kyoto Encyclopedia of Genes and Genomes; HP: Human Phenotype.

In addition to the PPi networks, an enrichment analysis was made using g:Profiler [37] (Table S5); Figure 6a shows the enrichment map of 103 proteins. The most significant ALT-related pathways from the enrichment analysis were correlated in a CIRCOS plot with the genes that showed the highest means of genomic alterations in the boxplot analysis displayed in Figure 3b,c.

The CIRCOS plot shows the interaction of the ALT-related genes in DNA repair, binding and metabolic processes; but, at the same time, the interaction of genes *RAD54B, NSMCE2, TERF1, SMG7, RECQL4, ATR, PRKDC, RAD1, RFC4,* and *NBN* is observed in telomere organization, maintenance and binding, HR, HDR, and HRR.

As mentioned earlier, pathways like HR and HRR are BIR-related processes, which are mechanisms used by ALT cells for telomere extension. The influence of mRNA up and down regulation and amplifications, as the dominant genomic alterations of the genes involved in these pathways, will be discussed later.

## 4. Discussion

TM is a crucial mechanism in the hallmarks of cancer for indefinite replicative potential, 10 to 15% of cancers do not depend on telomerase to maintain or extend its telomeres, instead, they use the ALT pathway [1]. In the last years, efforts to identify genes responsible for ALT progression have been made; so far, the main recurrent mutations identified in ALT+ cells are associated with *ATRX*/*DAXX* [1,15,39,40,41] *RAD51*/*RAD52* BIR-related dependent or independent pathways [1,6,7] and histone H3.3 [42]. Nevertheless, the ALT pathway mechanism and the molecular basis underlying its expression, switching, detection, and treatment remain elusive [43,44]; therefore, we used simplistic OncoOmics and in-silico approaches to identify potential molecular markers to improve the study of the ALT mechanism.

Commonly, ALT is enriched in tumors of mesenchymal origin [45], however, there is evidence of the coexistence of telomerase-mediated and ALT mechanisms in some solid tumors. In fact, some anti-telomerase-based treatments have shown the capability of some cells to switch to ALT and escape death [6,15]. Figure 4a shows the frequency of the ALT mechanism in the different PCA studies according to what has been reported; so far, a variation of ALT+ tumors in different types of cancer has been observed, for example, for UCS, Lee et al. 2011 reported 40% ALT+ cell lines [46], Heaphy et al. 2011 reported 7% and Lee et al. 2018 reported 0% [9]. On average, the following frequencies of ALT positive cell lines have been reported for the cancer types we found to have the highest frequencies of genomic alterations: OV 10% [8,9], UCEC 7% [8], ESCA 7% [8,9], BLCA 9% [8,9], ACC 18% [8,41], SKCM 25% [8,9,41,47], LUSC 4% [8,9,41], STAD 15% [9,41,48], and BRCA 3% [8,9,41]. Nonetheless, due to the difficulty and lack of sensible diagnostic techniques for ALT+ tumors, those numbers may fluctuate [12,49,50].

For our study, we found the highest frequency means of genomics alterations in the following PCA studies: UCS, OV, UCEC, ESCA, BLCA, ACC, SKCM, LUSC, STAD, and BRCA (Figure 3a). In addition, with the aid of boxplots, whose function is to show unusual values among a distribution of data, we identified 20 TM genes to be highly mutated among the 31 PCA studies (Figure 3b,c). This analysis, linked with the oncoprint showed in Figure 3d, correlates each altered gene with its main genomic alteration. As a result, *TP53* is the most mutated gene in the majority of cases, which is not odd, due to the evidence of its function as a tumor suppressor. Nonetheless, it has been reported to be co-mutated with *ATRX* and histone H3.3 [51], and its deletions are heavily related with abnormal expression patterns of *TERT* (a well-known factor for ALT progression) [52].

Along with *TP53*, additional ALT-related genes were identified and proposed in this study as possible molecular targets that have been predicted by Lovejoy et al. 2012 as potential elements of the ALT pathway like *UBR5, NSMCE2, RFC4, MCPH1, RECQL4, LRATD2, RAD1, RAD21, NBN, FBH1, RAD54B, ATR, SMG7,* and *TERF1* [42]. Likewise, according to Osterwald et al. 2015 and Chung et al. 2011, *SENP5, NSMCE2, NBN, ATR*, and TERF1 are involved in APBs formation [53,54], which are common elements of ALT+ cells for replication and extension of telomere ends [55]. Additionally, Dejardin et al. 2009 found *NSMCE2, RFC4, CCT5, NBN,* and *TERF1* to be expressed in the telomeres of ALT+ cell lines [56].

Some of the genes proposed have been predicted to play an important role in ALT-related mechanisms; for instance: *NSMCE2* recruitment is essential for APBs correct functioning [53], it is also part of the *SMC5/SMC6* complex, in which inhibition is known to disrupt APBs formation [57]. *NBN* is part of the MRN complex (*MRE11/RAD50/NBN*) that helps in the assemble of the G-overhang in the lead telomeric strand [58]; additionally, it promotes the ALT mechanism by recruiting *ATM* to the telomeres, allowing the invasion of adjacent telomeric DNA to be used as a template for telomere extension [59].

Moreover, *MCPH1* and *ARID1A* bind to the telomerase reverse transcriptase (hTERT) and regulate its expression [60]; the oncoprint in Figure 3d shows these genes to be down-regulated across different cancer types, this can give an insight into their role in the switching from a telomerase-mediated extension to ALT. Another gene, *LRATD2,* is known to be upregulated in cells with short telomeres [61]. *ATR* is important in the assembly of the telomerase complex [62]. *TERF1* is overexpressed in cells with long telomeres [63] and last but not least, *RECQL4* is associated with shelterin proteins POT1, TRF1 and TRF2 in the formation and stability of the t-loop and its knockdown causes telomere disfunction, possibly favoring the start of the ALT mechanism [64,65].

According to the TelNet database, based on their predicted activity in the ALT pathway, TM genes are classified as enhancers, repressors or ambiguous [19] (Figure 1). Only *NSMCE2, RFC4, NBN* and *ATR* are recognized as enhancers, while, *SENP5, UBR5, RAD21, MCPH1, RECQL4, ARID1A, CCT5, LRATD2, RAD1, FBH1, RAD54B, SMG7,* and *TERF1* are qualified as ambiguous. This highlights the importance of performing ex-vivo validations in the ALT-associated genes proposed in this study in order to improve the understanding of their role in the switching, progression or maintenance of ALT.

In addition to the 20 genes proposed for ex-vivo studies, we classified the genes based on their representation in the different types of ALT tumors proposed in Figure 4a. As shown in Figure 4b, there are genes altered only in determined groups of tumors. This can facilitate the development of future ex-vivo assays for specific types of cancers. For instance, the group of ALT-frequent tumors is represented by 20 genes like *NADK*, a kinase predicted to be a negative regulator of telomerase [66] or ERCC1, which is implicated in the processing and extension of the G-overhang [58].

In order to understand the role of the proteins in ALT+ cells, a protein-protein interaction network was performed using STRING (Figure 5a). The most significant ALT-related pathways (*p* < 0.001) were selected; 35% of the 103 proteins analyzed were involved in telomere organization, maintenance and telomeric DNA binding, while 22% are crucial for HR and HRR, which altogether with non-homologous end joining (NHEJ) have an important role in the mechanism by which ALT+ cells extend its telomeres [67,68].

Break-induced replication (BIR) is a specialized form of HR [7]. BIR is a recombination-dependent process that reinitiates DNA replication using a DNA homologue template, leading to a conservative DNA synthesis [7]. ALT is known to use a BIR bifurcated pathway dependent or independent of *RAD52*, both pathways relying on the activation of *POLD2* and *POLD3* [7]; however, the basis underlying this mechanism is still unclear [69]. Hence, to observe the interaction of the proteins proposed as potential targets in the study of ALT, a PPi network with the proteins of the HR pathway was constructed; additionally, the interaction evidence among the proteins is shown according to database curation, experimental determination and co-expression. Figure 5b shows that 10 of the proposed proteins are co-expressing (black lines) with the proteins in the HR pathway. All of the genes encoding for these proteins showed mRNA upregulation and amplification as their top genomic alterations in the oncoprint in Figure 3d. Gene amplifications can trigger the expression of genes in cancer cells, which could affect DNA repair and damage response [69,70], this could favor the upregulation of HR and BIR-related pathways in ALT+ tumors; nonetheless, further experimentation would be required to validate this statement.

Moreover, we performed an enrichment analysis for 103 proteins using g:Profiler, which searches a collection of proteins with pathways, networks, GO, and cancer phenotypes [71]. The GO for molecular functions were DNA binding and telomeric DNA binding; the GO for biological process were telomere maintenance, DNA repair, telomere, and chromosome organization. The most significant KEGG signaling pathway was HR, and the most significant REACTOME pathways were DDSB repair and HRR. Finally, the human phenotype related to the proteins analyzed was abnormality of chromosome stability (Figure 6a).

Furthermore, we selected the proposed ALT-related genes in our study, the most significant pathways from the PPi and enrichment analysis and constructed a CIRCOS plot (Figure 6b) to observe the main pathways in which they are involved. As anticipated, most genes like *RAD54B, NSMCE2, TERF1, SMG7, RECQL4, ATR, PRKDC, RAD1, RFC4,* and *NBN* are directly involved in pathways like DDSB, HR, HDR, and HRR among others, which are ALT-related processes. The enrichment analysis concluded that genes related to HR are upregulated and amplified in the PCA samples studied, which, as stated before, could be triggering the expression of ALT-dependent pathways.

In this study, we conducted a genomic analysis of 411 TM genes in 31 PCA studies and with the aid of different in-silico approaches, we proposed 20 candidate genes that are highly mutated as well as up and down regulated in different cancer types, which can be useful for future studies as potential molecular targets for the detection and understanding of the ALT pathway. Additionally, based on the prevalence of ALT+ tumors reported in this study, we strongly suggest to expand the study of ALT to a wide group of cancer types. Finally, we showed the need of a deep study of expression profiles and ex vivo assays to target TM genes that can help to understand the up and down regulation of ALT-related pathways and can provide guidance in the development of new therapies for the treatment of ALT+ tumors. We strongly believe this new decade will be promising for the study and understanding of this mechanism.

## Figures and Tables

**Table 1:** List of The Cancer Genome Atlas (TCGA) Pan-Cancer Studies with the number of individuals.

TCGA Study	*n*	TCGA Study	*n*
Acute Myeloid Leukemia (LAML)	165	Lung Squamous Cell Carcinoma (LUSC)	466
Adrenocortical Carcinoma (ACC)	76	Mesothelioma (MESO)	82
Bladder Urothelial Carcinoma (BLCA)	402	Ovarian Serous Cystadenocarcinoma (OV)	201
Brain Lower Grade Glioma (LGG)	507	Pancreatic Adenocarcinoma (PAAD)	168
Breast Invasive Carcinoma (BRCA)	994	Pheocromocytoma and Paraganlioma (PCPG)	161
Cervical Squamous Cell Carcinoma (CESC)	275	Prostate Adenocarcinoma (PRAD)	488
Cholangiocarcinoma (CHOL)	36	Sarcoma (SARC)	251
Colorectal Adenocarcinoma (COAD)	524	Skin Cutaneous Melanoma (SKCM)	363
Diffuse Large B-cell Lymphoma (DLBC)	39	Stomach Adenocarcinoma (STAD)	407
Esophageal Adenocarcinoma (ESCA)	181	Testicular Germ Cell Tumors (TGCT)	144
Glioblastoma Multiforme (GBM)	145	Thymoma (THYM)	119
Head and Neck Squamous Cell Carcinoma (HNSC)	488	Thyroid Carcinoma (THCA)	480
Kidney Renal Clear Cell Carcinoma (KIRC)	352	Uterine Carcinosarcoma (UCS)	56
Kidney Renal Papillary Cell Carcinoma (KIRP)	274	Uterine Corpus Endometrial Carcinoma (UCEC)	507
Liver Hepatocellular Carcinoma (LIHC)	348	Uveal Melanoma (UVM)	80
Lung Adenocarcinoma (LUAD)	503		

## Supplementary Materials

The following are available online at https://www.mdpi.com/2073-4425/11/7/834/s1, Table S1. Gene set. Gene set of TM maintenance genes related to ALT used in this study, Table S2. Genomic alterations. Total genetic alterations normalized by the number of samples observed in the 31 Pan-Cancer Atlas studies for the 411 TM genes, Table S3. Cancer stages. Number of genetic alterations normalized by the number of samples in each stage of the Pan-Cancer studies available, Table S4. Genomic alterations in PCA studies. Frequency means of genomics alterations for each of the 411 TM genes and the 31 Pan-Cancer Atlas studies, Table S5. Enrichment analysis. Enrichment analysis detailed results from g:Profiler, Table S6. TM genes classification in different groups of ALT tumors.
